# Characterization of human nasal organoids from chronic rhinosinusitis patients

**DOI:** 10.1242/bio.059267

**Published:** 2022-08-16

**Authors:** Mahnaz Ramezanpour, Harrison Bolt, Karen Hon, Gohar Shaghayegh, Hadi Rastin, Kevin Aaron Fenix, James Psaltis Alkis, Peter-John Wormald, Sarah Vreugde

**Affiliations:** 1 Department of Surgery-Otolaryngology, Head and Neck Surgery, Central Adelaide Local Health Network (Basil Hetzel Institute), The Queen Elizabeth Hospital and The University of Adelaide, Adelaide, Australia; 2 College of Medicine and Public Health, Flinders University, GPO Box 2100, Adelaide, South Australia 5001, Australia; 3 School of Chemical Engineering, The University of Adelaide, South Australia 5005, Australia

**Keywords:** Three-dimensional culture, Nasal organoid, Human nasal epithelial cells, Cilia, Goblet cell

## Abstract

Patient-derived organoids grown in three-dimensional cultures provide an excellent platform for phenotypic high-throughput screening and drug-response research. Organoid technology has been applied to study stem cell biology and various human pathologies. This study investigates the characteristics and cellular morphology of organoids derived from primary human nasal epithelial cells (HNECs) of chronic rhinosinusitis (CRS) patients. Nasal organoids were cultured up to 20 days and morphological, cell composition and functional parameters were measured by immunofluorescence, RT-qPCR, western blot and FACS analysis. The results showed that nasal organoids expressed the stem cell marker leucine-rich repeat-containing G-protein-coupled receptor 5 (LGR5), and markers for apical junction genes, goblet cells and ciliated cells. Moreover, we were able to regrow and expand the nasal organoids well after freezing and thawing. This study provides an effective and feasible method for development of human nasal organoids, suitable for the phenotypic high-throughput screening and drug response research.

## Abstract (precis)

**Summary:** This study provides an effective and feasible method for development of human nasal organoids from chronic rhinosinusitis patients, suitable for phenotypic high-throughput screening and drug-response research.

## INTRODUCTION

Chronic rhinosinusitis (CRS) is characterised by persisting inflammation of the mucosa of the nasal cavity and paranasal sinuses. CRS is a multifactorial disease associated with relapsing infections and causes a huge burden on health care systems (Bachert et al., 2014). To study the pathophysiology of CRS and host­–pathogen interactions, traditional cell culture techniques are used. These use primary human nasal epithelial cells (HNECs) grown in submerged monolayers or at air liquid interface (ALI) and have been instrumental in studying basic and applied aspects of airway biology, disease and therapy (Ramezanpour et al., 2019; Ramezanpour et al., 2018). Although both model systems have contributed enormously to the study of the pathophysiology of CRS, they also have their limitations. In order to get sufficient cells for experimental inquiry, HNECs have to be cultured for multiple passages, however, HNECs only retain their properties up to passage four, after which they go into senescence (Muhammed et al., 2015). Moreover, as for any primary cell, HNECs vary in their response to different challenges and thus experiments have to be repeated using cells from different donors. HNECs cultured at ALI differentiate and form a unique cell culture system that is more representative of the airway mucosa and is particularly well suited to study the mucosal barrier structure and function. However, it takes up to 6 weeks for HNEC-ALI cultures to become mature and experiments testing challenges applied to their apical surface can be conducted only once, after which cells need to be harvested or discarded (Park et al., 2016; Ramezanpour et al., 2019).

Recent advances in cell culture methods have resulted in the development of ‘organs in a dish’, where organoids self-organise into a three-dimensional (3D) structure with phenotypic and functional traits analogous to the original biological specimens (Simian and Bissell, 2017). Organoid technology may address many of the limitations of traditional cell culture methods. Unlike cell culture work, organoid growth only requires small amounts of biopsy material or nasal brushings as cultures can be expanded to millions of cells. This can provide sufficient replicates for moderate-to-high-throughput applications. 3D imaging (organoid) is superior in visualising the complexity of biological specimens compared with 2D imaging (ALI culture); therefore, nasal organoids may provide better understanding of cell shape and cellular composition (Dekkers et al., 2019; Farahani et al., 2017). Another promising direction is the creation of organoid biobanks. Since organoids can in theory withstand freezing-thawing cycles, they can be stored in biobanks for screening new therapeutic drugs and studying the heterogeneous responses of CRS patients to standard treatment. Organoid models have become beneficial in development of personalised medicine (Liu et al., 2020) and modelling infectious diseases (Bartfeld and Clevers, 2015). The generation of organoid models has been extended to a wide range of organs such as the intestine (Sato et al., 2009), breast (Sachs et al., 2018), brain (Hattori, 2014), prostate (Karthaus et al., 2014) and salivary gland (Maimets et al., 2016). Inspired by those 3D cell culture systems, we describe the development of an *in vitro* nasal organoid model derived from patient samples (nasal brushes). Each organoid recapitulates the highly differentiated airway epithelium and forms a self-organised pseudostratified epithelium that contains goblet cells and ciliated cells. This culture technique provides an alternative method for *in vitro* human airway that does not require cell culture inserts, thus facilitating high-throughput culture. Brewington et al. (2018) and Liu et al. (2020) described the development of nasal epithelial cell organoids models from cystic fibrosis patients. Cilia, mucins, tight junctions and the cystic fibrosis transmembrane conductance regulator (CFTR) have been studied in the nasal CF model (Liu et al., 2020; Brewington et al., 2018). Our study extends on these findings by thoroughly characterised the morphological, cell composition, and functional parameters of nasal organoids in CRS patients.

## RESULTS

**Fig. 1.**
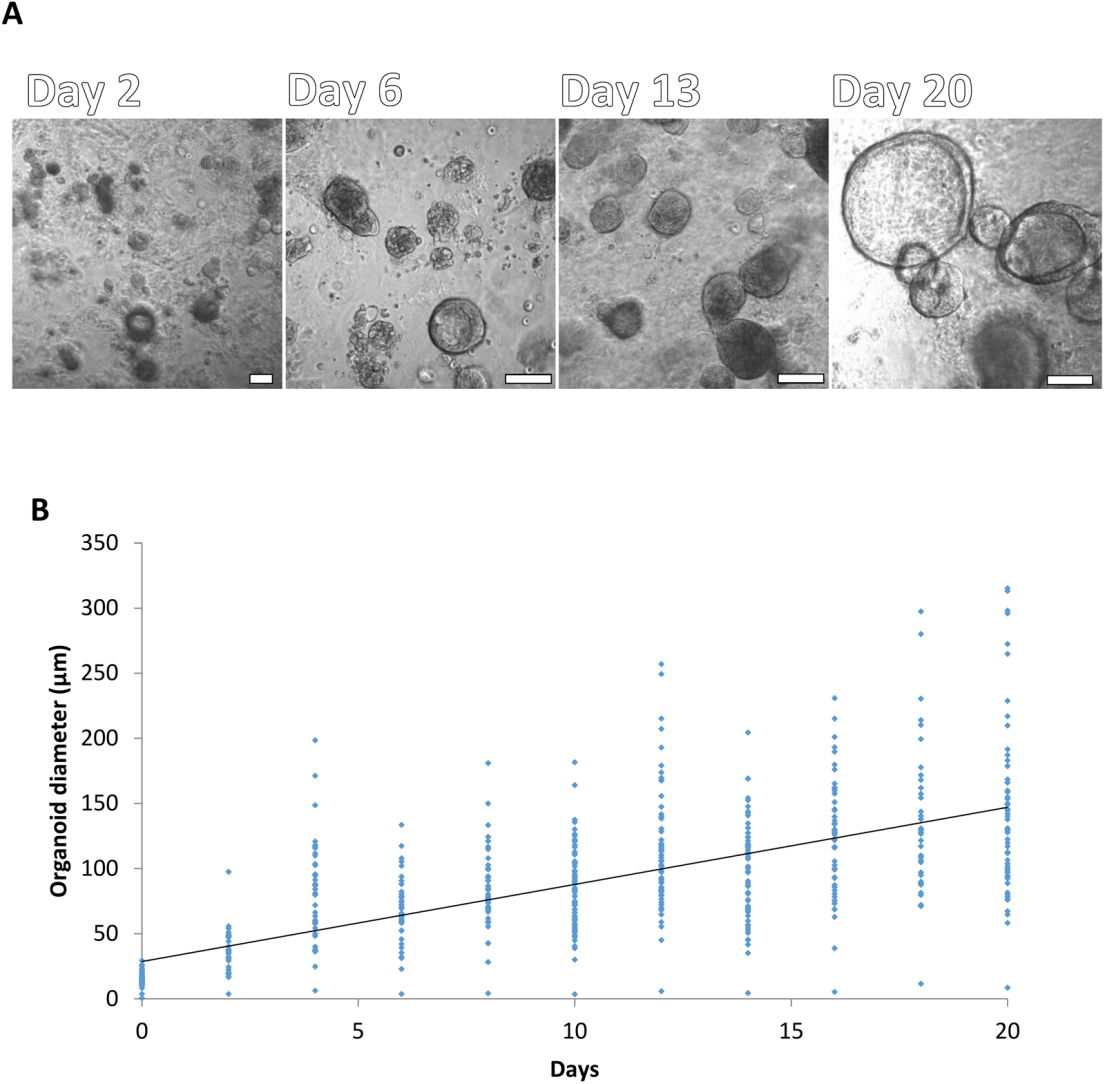
**Morphology of nasal organoids generated from primary human nasal epithelial cells.** (A) Images were taken from a different area of each well at different time points 2, 6, 13 and 20 days (from left to right respectively). The white bar is 100 µm and 20x objective. (B) The area of organoids was measured by Image J software and converted into length. For each well, between 30 to 80 organoids were imaged (B). Data presented as mean±standard deviation (s.d.), *n*=6.

HNECs were grown into organoids from a total of six donors (four males and two females, aged 30–73 years). HNECs appeared to grow into organoids within 1 week and expanded in size over a period of 20 days (Fig. 1). Around day 6-13, a lumen became visible within some of the organoids and by day 20 almost all organoids demonstrated a lumen (Fig. 1A).

Fig. 1B shows the size distribution of organoids (six donors) grown from day 2 to day 20. After day 10, the average diameter of organoids was between 100 µm and 150 µm. A significant positive correlation was found between size and time. The linear regression was performed and the *P*-value was less than Pearson correlation, *P*<2.2×10^−16^ (Fig. 1B).

### Biobanking of nasal organoids

**Fig. 2.**
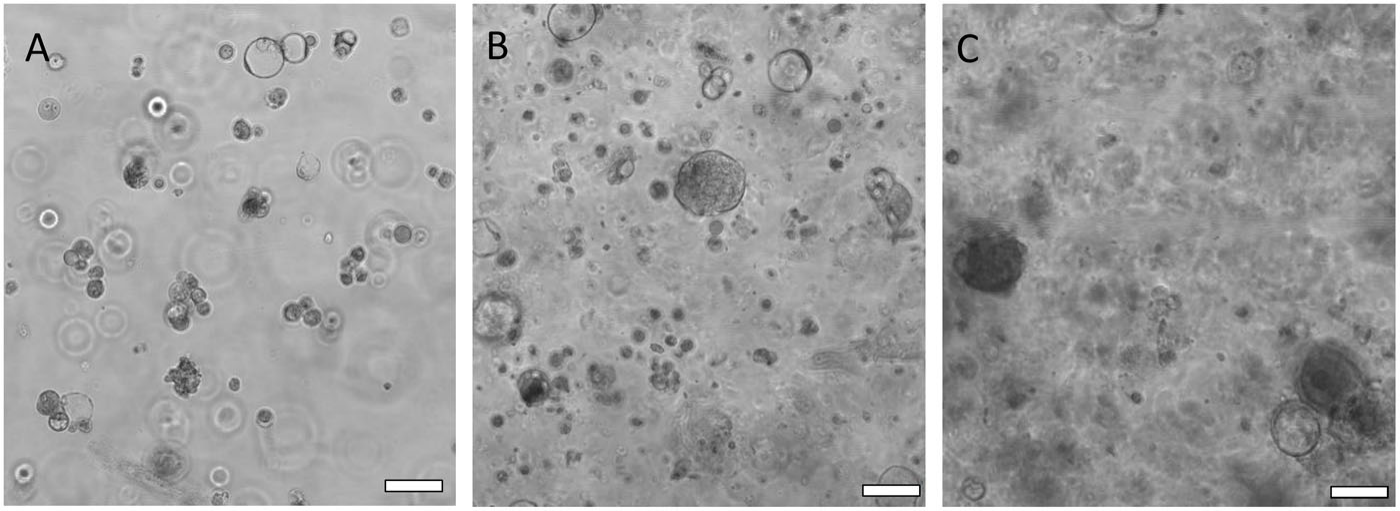
**Nasal organoids recover after thawing.** Nasal organoids from primary human nasal epithelial cells (HNECs) 3 days post-thawing (A); after 7 days, organoids appear cystic (B); at 10 days, organoids appear dense and solid (C). PMT images of organoids at 20x magnification using a Zeiss 700 LSM confocal microscope. Scale bar: 20 µm and 20x objective.

To investigate whether HNEC organoids can freeze, thaw and be re-established, we grew organoids from the frozen stock. Our results showed that the nasal organoids expanded rapidly and could be frozen and thawed similarly as cell lines. Fig. 2A shows three days post-thaw nasal organoids that were small but healthy-looking (Fig. 2A). After 7 days, organoids were more cystic and appeared hollow (Fig. 2B). Fig. 2C shows 10-day-old organoids in differentiated media where they keep getting bigger and more complicated after 10 days (Fig. 2C).

### Stem cell marker *Lgr5* is highly expressed in nasal organoids

**Fig. 3.**
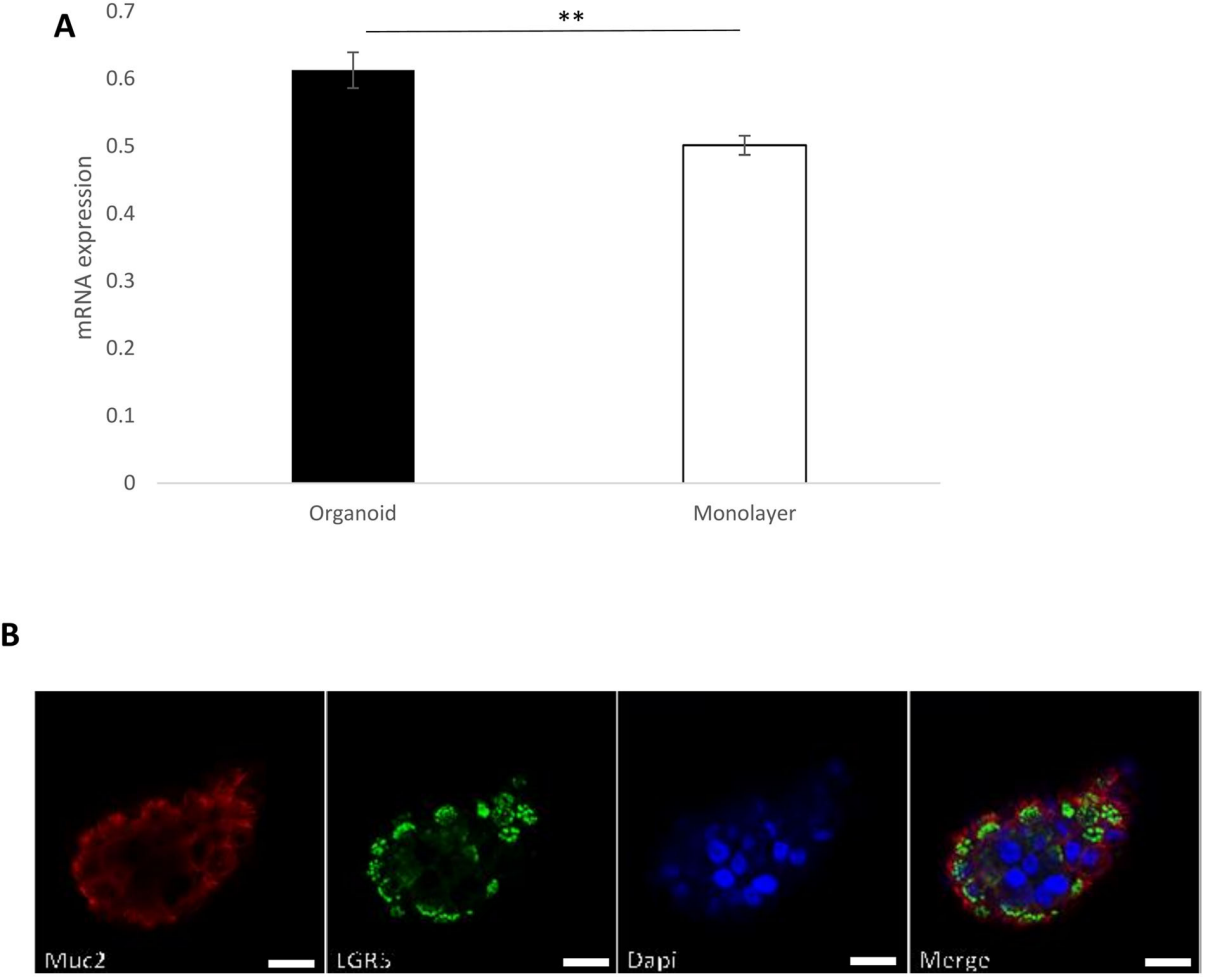
**LGR5 is highly expressed in nasal organoids.** The stem cell marker Lgr5 mRNA expression levels in organoids and monolayer cultures, GAPDH was used as a housekeeping gene and data were normalised to GAPDH. The values are shown as means±s.e.m., *n*=5, ** *P*=0.001 (A). Representative immunofluorescence of 10–12-day-old organoid showing expression of MUC2 (mucus secreting cell, red) and Lgr5 (stem cell marker, green). DAPI stains nuclei blue (B), Scale bar: 20 µm and 20x objective.

As human organoids derived from leucine-rich repeat-containing G-protein-coupled-receptor-5 (*LGR5*)-positive adult stem cells represent a new cell source for regeneration (Schneeberger et al., 2020), we analysed the mRNA expression level of the stem cell marker *Lgr5* in the organoid and monolayer culture using HNECs from five donors, grown for 10-12 days. Organoids showed a significant increase in *Lgr5* mRNA expression (*P*=0.001) compared with that of same age monolayer cultures (Fig. 3A). In line with the mRNA expression, the organoids showed positive expression of Lgr5 protein by immunofluorescence assay (Fig. 3B).

### Characterisation of nasal organoids composition

**Fig. 4.**
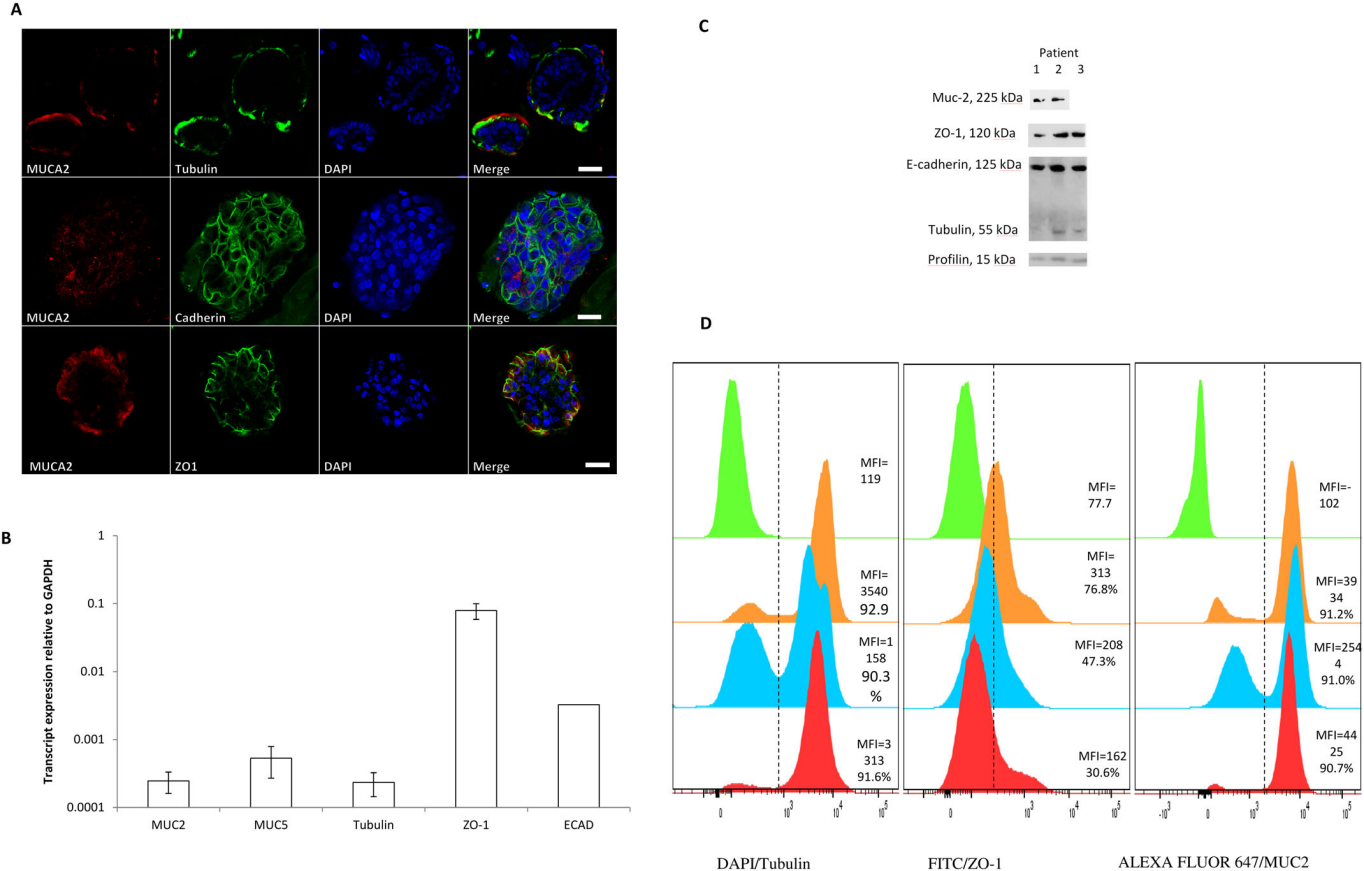
**Characterisation of nasal organoids composition.** Organoids grown from human nasal epithelial cells (HNECs) from CRS patients were stained for goblet cells: (MUC2); cilia: (Tubulin) and apical junction proteins: (E-cadherin and ZO-1). Scale bar: 20 µm and 20x objective. (A) Mean absolute quantification of differentiation markers (Muc2, Muc5, Tubulin, Zo-1 and E-cadherin) expression by absolute quantification assay in 12 days cultured nasal organoids from four donors. The standard curve was generated by real time PCR amplification using as template increasing dilutions of a PCR product of the organoids; GAPDH was used as a housekeeping gene and data were normalised to GAPDH (B). Western blot analysis of MUC2 (225 kDa), E-cadherin (125 kDa), ZO-1 (120 kDa) and Tubulin (55 kDa) for nasal organoids from CRS patients, Profilin-1 (15 kDa) was used as a loading control (C). Mean fluorescence intensity (MFI) and percentage of positive populations have been shown (D).

The airway epithelium is composed of five cell types including basal, goblet, ciliated, club, brush cells and cells of the diffuse neuroendocrine system (Carlier et al., 2021) To determine whether our LGR5+ organoids cultured on day 12 formed a microarchitecture mimicking the nasal mucosa *in vivo*, we investigated their expression of functional molecules characteristic of nasal tissues using immunofluorescence, mRNA expression, western blot and FACS analysis. Firstly, we examined the expression of markers for goblet cells (Muc2), cilia (Tubulin) and apical junctional complexes (E-cadherin and ZO-1) by immunofluorescence. Human nasal organoids had positive staining for all of those markers (Fig. 4A). Secondly, we investigated the mRNA expression of differentiation markers (*Muc2, Muc5, Tubulin, ZO-1* and *E-cadherin*) in nasal organoids by absolute quantification of qPCR. There was positive, variable expression of *Muc2, Muc5, Tubulin* and higher levels of expression of *ZO-1* and *E-cadherin* (Fig. 4B). Thirdly, in line with immunofluorescence and absolute quantification results, the western blot results showed the presence of bands specific for MUC2 (225 kDa), E-cadherin (125 kDa), ZO-1 (120 kDa) and Tubulin (55 kDa) in nasal organoids (Fig. 4C). Next, we assessed the organoids with FACS analysis. Representative flow cytometry gating strategy for MUC2, Tubulin and ZO-1 expression in nasal organoids are shown in Fig. S1. MUC2, Tubulin and ZO-1-specific mean fluorescence intensity (MFI) were more than 91%, 90% and 50% respectively, compared with fluorescence minus one (FMO) (Fig. 4D). Finally, by 4 weeks, the cilia were functional as evidenced by time-lapse microscopy showing beating cilia in Movie 1.

**Fig. 5.**
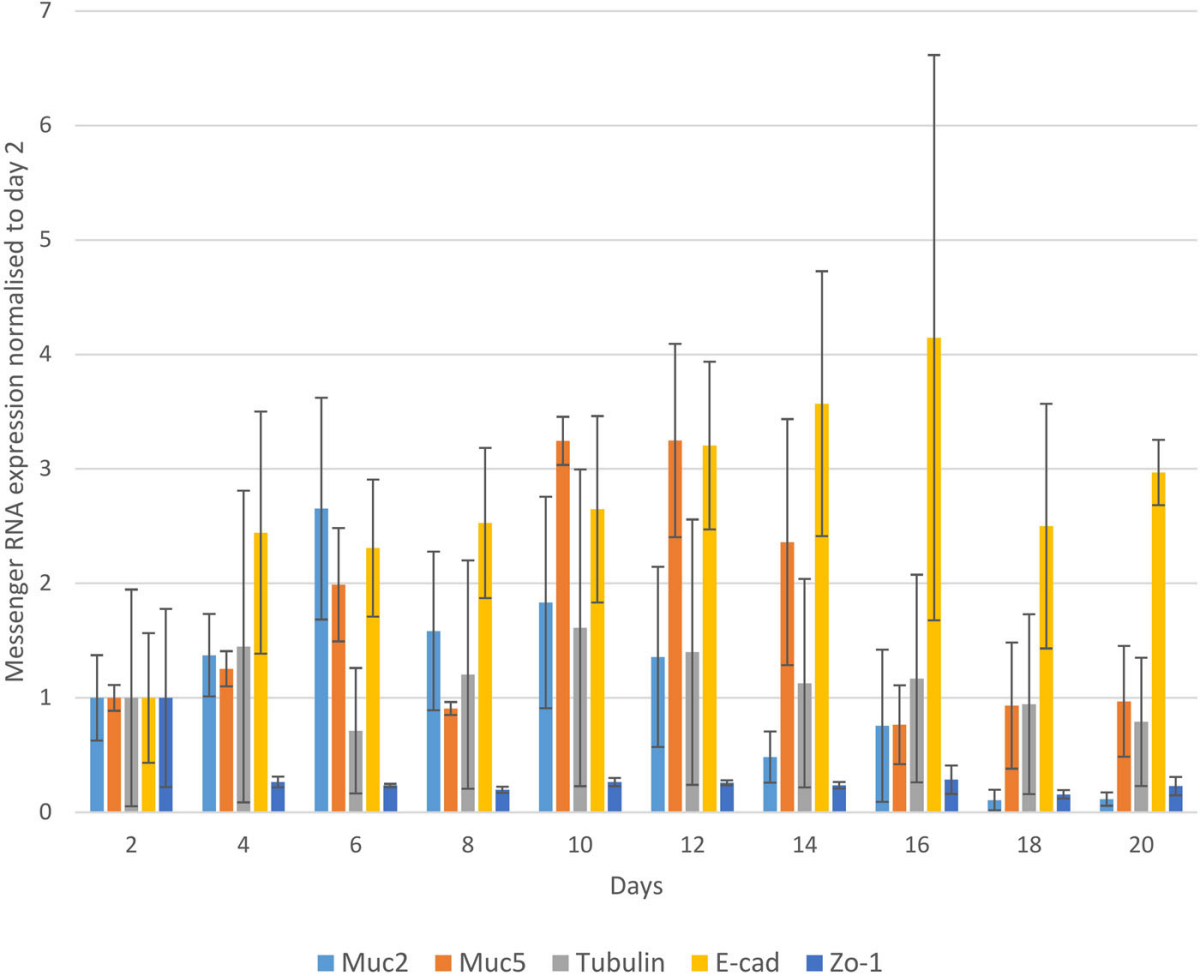
**The nasal organoids differentiation**. Nasal organoids were characterised to verify the presence of differentiation markers (Muc2, Muc5, Tubulin, E-cadherin and ZO-1) for 20 days by qPCR. For the qPCR, each time point for each gene represents experimental triplicates from three CRS patients. GAPDH was used as a housekeeping gene and data were normalised to GAPDH.

To characterise organoids over time, we collected the organoids every second day and the mRNA expression of *Muc2*, *Muc5*, *Tubulin*, *E-Cadherin* and *ZO-1* was measured up to day 20 (from three donors) (Fig. 5). The expression of *Tubulin* and *MUC5* gradually increased and were highest at around day 10 to day 12. *Muc2* showed the highest expression at day 6, after which expression gradually reduced, particularly at and beyond day 14, but this did not reach statistical significance (*P*>0.05). *E-cadherin* expression gradually increased with a peak reached at day 16 whilst expression of *ZO-1* remained low across all time points beyond day 2 (Fig. 5). Organoids were immunolabeled for Claudin, Tubulin, MUC2, ZO-1 and DAPI and visualised using confocal microscopy. We observed the gradual increase of all markers (Claudin, Tubulin, MUC2 and ZO-1) with changes in subcellular localisation over the time (Fig. S2A and B).

## DISCUSSION

In this study, we generated nasal organoids from CRS patients that could self-renew for many generations and appeared to exhibit a centralised lumen surrounded by a polarised airway epithelial cell layer, comprised of differentiated cell types. Nasal organoids expressed *Lgr5*, a known adult stem cell marker (Leushacke and Barker, 2012; Wu et al., 2013), and contained mucus secreting cells and ciliated cells. Organoids formed apical junctional complexes after approximately 12 days with a cilia functional system evident after 1 month.

Nasal epithelium patient-derived cell culture models are required as representative systems to support studies into CRS disease pathophysiology, host–pathogen interactions and for the preclinical development of new therapeutic approaches. For years, the majority of efforts to differentiate nasal airway epithelial cells into representative models have focused on air-liquid interface (ALI) cultures (Ramezanpour et al., 2017, 2020). However, the requirement of porous culture inserts limits the application of ALI culture to smaller-scale experiments. In contrast, nasal organoids bring the possibility to perform high-throughput screening (Phan et al., 2019; Mead et al., 2020 preprint). Furthermore, evaluation of the expression levels of the mesenchymal stem cell markers CD73, CD90, CD105 and the hematopoietic stem cell marker CD45 in HNECs (monolayers) showed that the quality of the cells was maintained up to passage four (Muhammed et al., 2015). In contrast, HNECs when cultured under 3D-spheroid-forming conditions can be maintained for many generations. Importantly and in contrast to HNEC-ALI cultures, nasal organoids can be frozen and thawed, thereby significantly expanding the scope and potential of such culture techniques. *Lgr5*+ cells in the respiratory epithelium are proliferative stem/progenitor cells and they participate in the regeneration of lesioned nasal respiratory epithelium (Zhang et al., 2016; Ren et al., 2021). Previous studies showed the expression of *Lgr5* in mice nasal respiratory epithelium (Zhang et al., 2016). Here, we compared the expression of the stem cell marker Lgr5 between the nasal organoids and monolayer culture. To our best knowledge, this is the first study showing the expression of the stem cell marker Lgr5 in human nasal epithelial cells. Some studies showed generation of nasal organoid model of CFTR function and modulation using primary HNEs however, the model was limited by low throughput (Brewington et al., 2018). Our study addressed this problem by making a bio-bank of the matched samples therefore we are able to expand the potential of organoid techniques. We characterised the morphological, cell composition, and functional parameters of nasal organoids for 20 days by different assays. Further studies are required to compare the properties and phenotype of CRS versus non-CRS control organoids with respect to mucociliary function, mucus production and tight junction integrity.

In conclusion, we have established and characterised nasal organoids derived from CRS patients and demonstrated their morphological changes and associated gene expression patterns over time. They provide a physiologically relevant experimental model and platform for pharmaceutical research, investigating host-pathogen interactions, and disease pathophysiology in the context of CRS.

## MATERIALS AND METHODS

### Primary human nasal epithelial cells

Our study was performed at the Central Adelaide local health network human research ethics committee (CALHN HREC) (HREC/18/CALHN/69), with ethics approval and written consent obtained from patients prior to the collection of primary human nasal epithelial cells (HNECs). HNECs were obtained from the inferior turbinate surface with sterile nasal brushes from CRS patients who were undergoing endoscopic skull base surgery. Nasal brushings were suspended in nasal epithelial growth media (STEMCELL Technologies Australia Pty. Ltd, Tullamarine, VIC, Australia). Extracted cells were then depleted of monocytes using anti-CD68 (Dako, Glostrup, Denmark) coated culture dishes. HNECs were expanded in T-25 flasks (ThermoFisher Scientific, Waltham, MA, USA) in routine cell culture conditions of 37^∘^C humidified air with 5% CO_2_ in collagen-coated flasks (ThermoFisher Scientific) as a monolayer culture.

### 3D culture of nasal epithelial cells and organoid passage

First, 40% Matrigel solution (Corning Matrigel Matrix, NY, USA) was prepared by promptly mixing the cold PneumaCult Airway Organoid Seeding Medium (STEMCELL Technologies) and cold Matrigel (Corning, MA, USA). Then 500 μl 40% Matrigel solution aliquot per well of a 24-well plate was added and the plate incubated at 37°C for 30 min to allow the Matrigel layer to solidify. Meanwhile, HNECs were washed twice with 2 ml PBS and 2 ml 0.025% Trypsin-EDTA was added to each flask. The cells were incubated at 37°C for 3-5 min, until the cells dislodged with gentle tapping of the flask. The cells were neutralised with 5% foetal bovine serum (FBS, Gibco, Life Technology, USA) in PBS and centrifuged at 300× ***g*** for 5 min. The cell pellet was resuspended in 2 ml 5% Matrigel solution in the airway organoid seeding medium. The cells were prepared at a concentration of 180,000 cells/ml in 5% Matrigel solution and 500 μl cell solution was plated per well of a 24-well plate (approximately 50,000 cells per cm^2^). The cells were incubated at 37°C, 5% CO2 in a humidified incubator. In the differentiation step, the medium replaced with airway organoid differentiation medium (STEMCELL Technologies). Medium changes were performed three times per week by gently aspirating the medium above the semi-solid Matrigel layer and replacing with 500 μl fresh 5% Matrigel solution in airway organoid differentiation medium.

For subculture, the 5-7 days old organoids (high density) were passaged using cell recovery solution (Corning, MA, USA). Briefly, cultured organoids were washed with cold PBS, and 500 µl cold cell recovery solution was added to the wells and incubated for 20 min at 4°C. After washing with PBS, dissociated organoids were passaged at a ratio of 1:5. Freshly prepared medium and Matrigel were then added for the organoid culture. The passage of organoids cultured under medium was performed once per week until the indicated passage.

### Freezing and thawing of *in vitro* cultured organoids

For organoid cryopreservation, first the organoids (5-7 days old) collected from up to 12 wells of 24-well plates into one 15 ml tube and centrifuged at 4°C, 300× ***g*** for 5 min. The supernatant was removed and cold cell recovery solution (Corning, MA, USA) was used to dissociate the organoids into single cells. After centrifugation (4°C, 300× ***g*** for 5 min), the pellets (2×10^6^ viable cells) were resuspended in 500 μl of freezing medium (Cryostor CS10, Stemcell Technologies, Cambridge, UK). Samples were slowly frozen by placement in a Styrofoam container in a −80°C freezer. Frozen samples were thawed in a 37°C water bath, and thawed organoids were then cultured as detailed above.

### Immunofluorescence microscopy

The top medium from the well was removed without disturbing the Matrigel layer. Then 500 µl of cold cell recovery solution was added to the culture for 20 min on ice. After breaking the Matrigel by pipetting, the organoids were transferred into a new Falcon tube and centrifuged at 300× ***g*** for 5 min at 4°C. The pellet was re-suspended in 100 μl airway organoid seeding medium. Cytospin 4 was then used at 800 rpm, low acceleration for 2 min (ThermoFisher Scientific) to attach the organoids to the cytospin slides. The organoids were then fixed with 2.5% formalin in phosphate-buffered saline (PBS) for 10 min. Fixed samples were permeabilised with 0.1% Triton X-100 in PBS for 15 min, blocked for 1 h with serum-free protein block (SFB; Dako, Glostrup, Denmark), and incubated with mouse LGR5 monoclonal antibody (MA5-25644, Invitrogen, Carlsbad, CA, USA), rabbit Anti-MUC2 antibody (ab90007, Abcam, Cambridge, UK), mouse anti-alpha Tubulin antibody (ab7750, Abcam, Cambridge, UK), mouse Anti-E-cadherin (610182, BD BioSciences, CA, USA) or mouse monoclonal Anti-human Zo-1 (339100, Invitrogen) overnight at 4°C. In negative controls, the primary antibody was replaced with PBS. Excess primary antibody was removed, and 2 μg/ml anti-Rabbit CY3 or 2 anti-mouse Alexa-594 conjugated secondary antibody (Jackson ImmunoResearch Labs Inc., West Grove, PA, USA) were added and incubated for 1 h at RT. The samples were rinsed in TBST, and after the third wash, 200 ng/ml of 4′, 6-diamidino-2-phenylindole (DAPI; Sigma-Aldrich, USA) was added to resolve nuclei. Membranes were transferred to a glass slide and a drop of anti-fade mounting medium (Dako, Glostrup, Denmark) was added before cover-slipping. Samples were visualised by using an LSM700 confocal laser scanning microscope (Zeiss Microscopy, Germany).

### Western blotting of organoid proteins

The proteins from organoid cultures were measured for concentration using a BCA protein assay kit (catalogue number 23337, ThermoFisher Scientific, IL, USA). Samples were separated by 4% to 12% Bis-Tris Gel (Novex, Life technology, San Diego, CA, USA) and were transferred onto nitrocellulose membranes (Novex). Blots were probed with primary antibodies: mouse anti-Zo-1 [1:1000], mouse anti-alpha Tubulin [1:1000], mouse anti-E-cadherin [1:2500], rabbit anti-Muc2 [1:1000] and rabbit polyclonal anti-human profilin I antibody (1:10,000; ThermoFisher Scientific). Secondary antibodies used were HRP-conjugated goat anti-rabbit (Abcam plc, Melbourne, Australia) and goat anti-mouse (Abnova Corporation, Taipei City, Taiwan). Protein bands were developed using Invitrogen ECL chemiluminescent substrate (ThermoFisher Scientific). Luminescence was detected using the LAS–4000 Imager (Fugifilm, Tokyo, Japan).

### RNA extraction, reverse transcription and absolute quantification of qPCR

Total RNA was extracted from the organoid samples using the Qiagen RNeasy Mini kit (Qiagen GmbH, Hilden, Germany) according to the manufacturer's instructions followed by DNAse treatment with RNase-Free DNAse set (Qiagen). Extracted RNA was assessed for quality using the Experion RNA StdSens analysis kit (Bio-Rad Laboratories, Hercules, CA, USA) and total quantification using the Nanodrop 1000 spectrophotometer (ThermoFisher Scientific). RNA was reverse transcribed into cDNA using Quantitect Reverse Transcription kit (Qiagen, Hilden, Germany) with a MyCycler Thermal Cycler (BioRad Laboratories Inc., Gladesville, Australia). The resulting cDNA was subjected to qPCR with TAQman primer/probe sets for each target gene, Taqman Universal Master Mix II (ThermoFisher Scientific, Scoresby, Australia) and nuclease-free water. We quantified PCR products using a Qubit 3 Fluorometer (ThermoFisher Scientific, MA, USA) and generated a standard curve of the absolute quantification with a series of five 1:10 dilutions of qPCR products. The expected amplicon size was 75-148 bp and the primer sequences are listed in Table 1. Taqman gene assays used for gene expression analysis were: LGR5 Hs00969422_m1 (*Lgr5*), Hs01023895_m1 (E-cadherin-1), Hs01551861_m1 (Zo-1), Hs01095754_m1 (tubulin-alpha-1a), Hs01370716_m1 (Muc2), Hs00894040_m1 (Muc5) and Hs02758991 (GAPDH). A melt curve performed at the end of the amplification was undertaken to confirm that there was only a single product amplified in each reaction. The data were normalised to GAPDH as a reference gene.

**Table 1.**
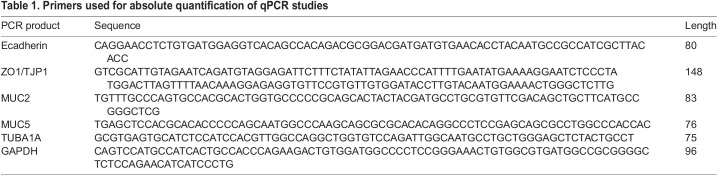
Primers used for absolute quantification of qPCR studies

### Flow cytometry

The total cells were determined after breaking the Matrigel using cold cell recovery solution and centrifuged at 300× ***g*** for 5 min at 4°C. To exclude dead cells, cells were stained with Fixable Viability Dye eFluor 780 (BD Bioscience, CA, USA) for 15 min at RT. After centrifugation, the cells were blocked with Fc receptor binding inhibitor monoclonal antibody (BD Bioscience, CA, USA) in Flow Buffer (PBS+2% FBS+2 mM EDTA) for 10 min at RT. Surface staining of the cells was performed by incubating with Anti-ZO1 tight junction protein antibody [mAbcam 61357] (FITC) (ab150266, Cambridge, UK) for 30 min on ice. After surface staining, the cells were fixed and permeabilised for 20 min at room temperature using BD Cytofix/Cytoperm buffer (BD Biosciences, CA, USA) and washed twice with the BD perm/wash buffer. Intracellular staining was then performed by incubating with MUC2 Antibody (996/1) [Alexa Fluor 647] and alpha Tubulin Antibody (DM1A) [Alexa Fluor 405] (Novous, CO, USA) on ice for 15 min (Table 2). The cells were washed three times with BD perm/wash buffer and analysed by BD FACS Canto II instrument (BD Bioscience, CA, USA). The data were analysed by FlowJo software package (TreeStar, USA). Acquired data was gated to include only single cells (FSC-W/FSC-H and SSC-W/SSC-H) that were alive (FSC-A/APC-Cy7). Positive cellular expression of markers was gated based on fluorescence minus one (FMO) controls.

**Table 2.**
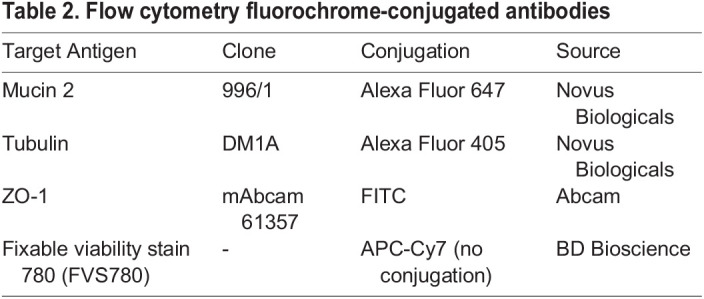
Flow cytometry fluorochrome-conjugated antibodies

### Measurement of organoids size

Organoids formed from HNECs were imaged using a LSM700 confocal laser scanning microscope (Zeiss Microscopy, Jena, Germany). The images of the organoids were analysed using ImageJ software (National Institutes of Health, Bethesda, MD, USA). Organoids were visualised every second day from day 0 to day 20. All images were converted to simplified threshold images under the same converting condition and the edges of the organoids were then detected using a selection tool. Feret's diameters of the detected spheroid edges were measured initially as pixels and converted to micrometres by comparing to a reference length (Dražić et al., 2016).

### Cilia function and structure

Ciliary beat frequency (CBF) of organoid culture was assessed using a 20X objective, and 1.5× magnification on an inverted microscope (Olympus IX70, Tokyo, Japan). Video was recorded using a Model Basler acA645-100 μm USB3 camera (Basler AG, Ahrensburg, Germany) at 100 frames per second at a resolution of 640×480 pixels. The recorded video samples were analysed using the Sisson-Ammons Video Analysis (SAVA) system.

### Statistical analysis

Absolute transcript copy numbers for each gene and replicate were calculated with the ViiA7 system (Applied Biosystems, CA, USA). Transcript copy numbers were normalised against the housekeeping gene GAPDH. The analysis was performed using one way analysis of variance followed by Tukey's honestly significant difference post-hoc test using IBM SPSS Statistics 25. Organoid sizes were measured by imageJ and the Pearson correlation test and linear regression were performed. Data are presented as mean±standard error of the mean (s.e.m.). A *P*-value less than 0.05 was considered statistically significant.

## Supplementary Material

Supplementary information
