# Supplementary material for: Characterization of human nasal organoids from chronic rhinosinusitis patients
Source: Biol Open. 2022 Aug 16;11(8):bio059267. doi: 10.1242/bio.059267 (PMC9399817; doi:10.1242/bio.059267)
Supplement: Supplementary information [file biolopen-11-059267-s1.pdf]

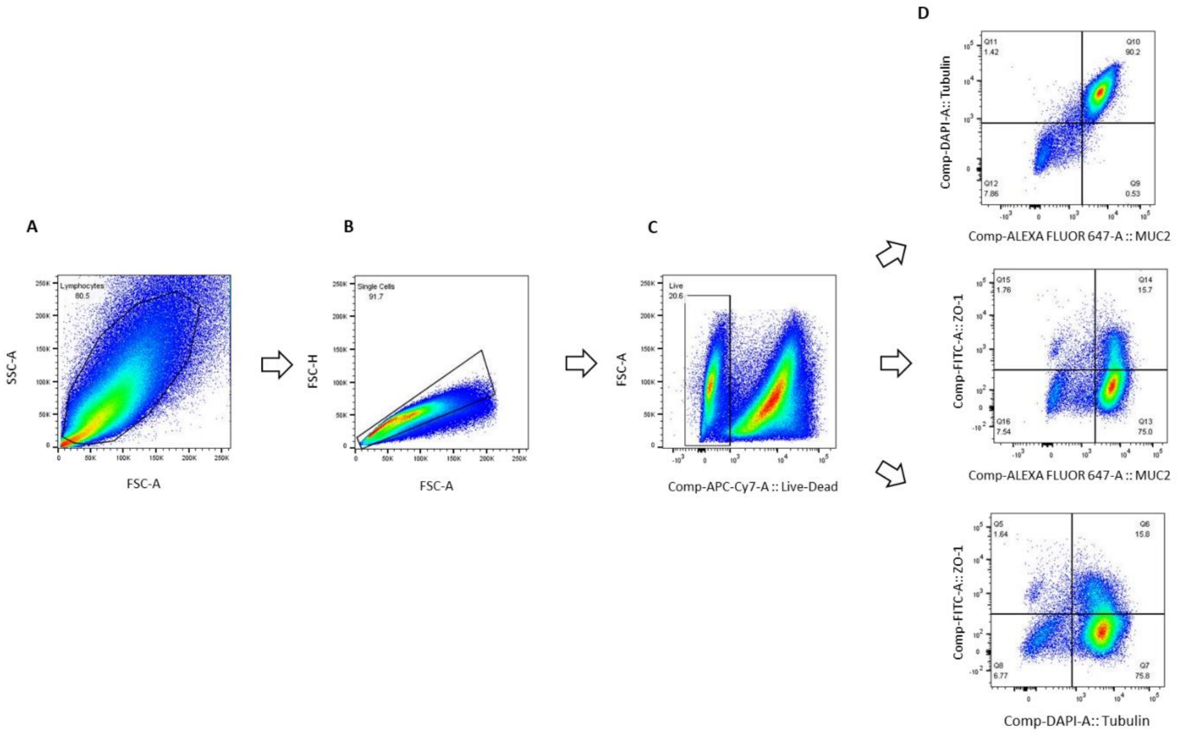

Fig. S1. Representative flow cytometry gating strategy for MUC2, Tubulin and ZO-1 expression in nasal organoid model. The organoid population was gated according to forward- and side-scattered properties (FSC-A & SSC-A) (A); Doublets were removed according to FSC-A and FSC-H (B); Live cells were gated according to FSC-A and APC-Cy7 (C); MUC2+, Tubulin+ and ZO-1+ cells were gated using anti-MUC2 ALEXA FLUOR 647, anti-Tubulin DAPI and anti-ZO-1 FITC antibodies respectively (D).

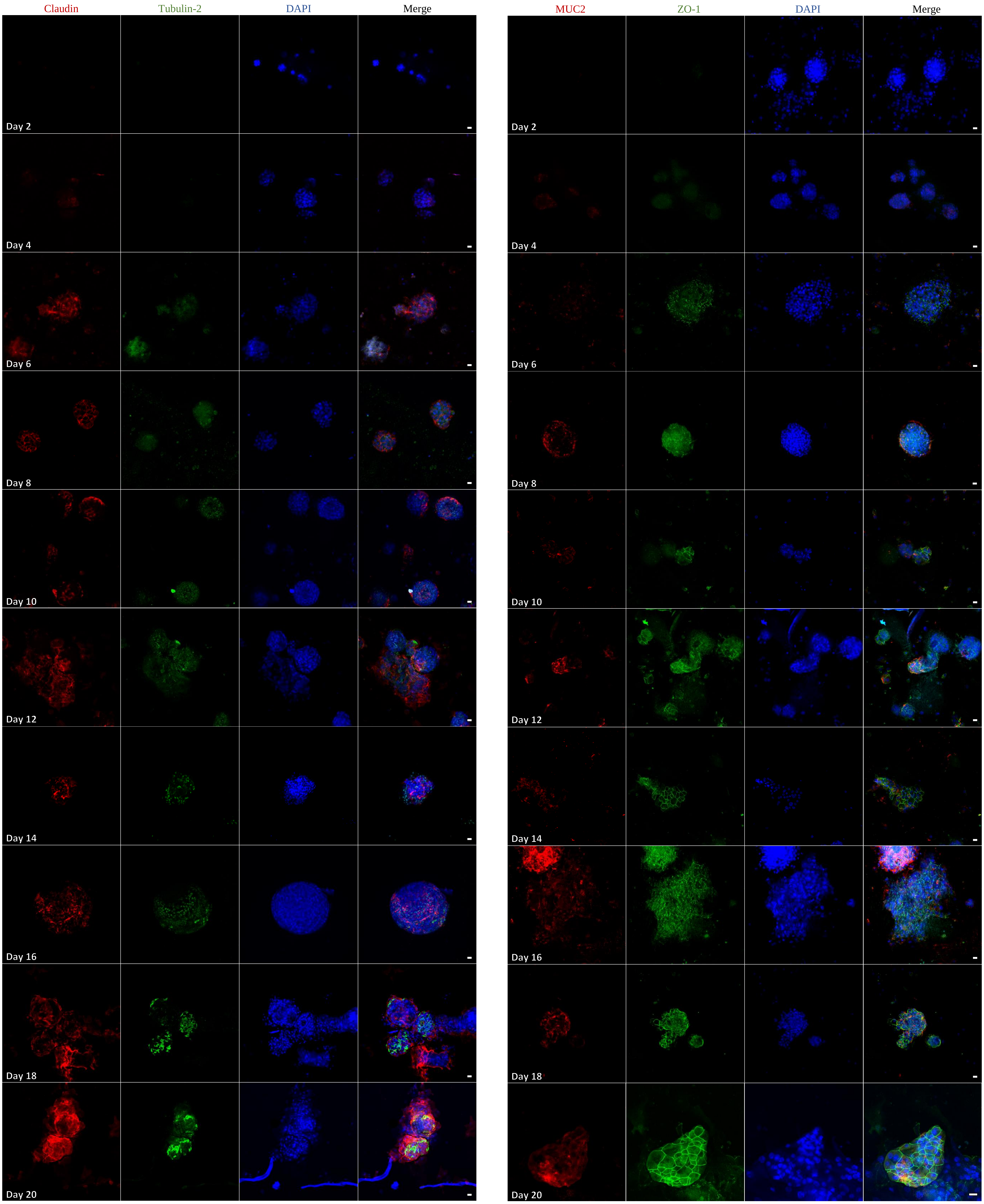

Fig. S2. The nasal organoids differentiation. Nasal organoids were characterized to verify the presence of differe ntiation markers (Muc 2, Muc5, Tubulin, E-c ad and ZO-1) for 20 days by immunofluorescence assay (A&B). 3D confocal image of a human nasal epithelial organoid immunolabeled for Claudin (red), Tubulin (green), MUC2 (red), ZO-1 (green) and DAPI (blue). Scale bar 20 µm and 20× objective. All images are representative images observe d from within at least three organoids in this particular experiment (A&B).

Biology Open • Supplementary information

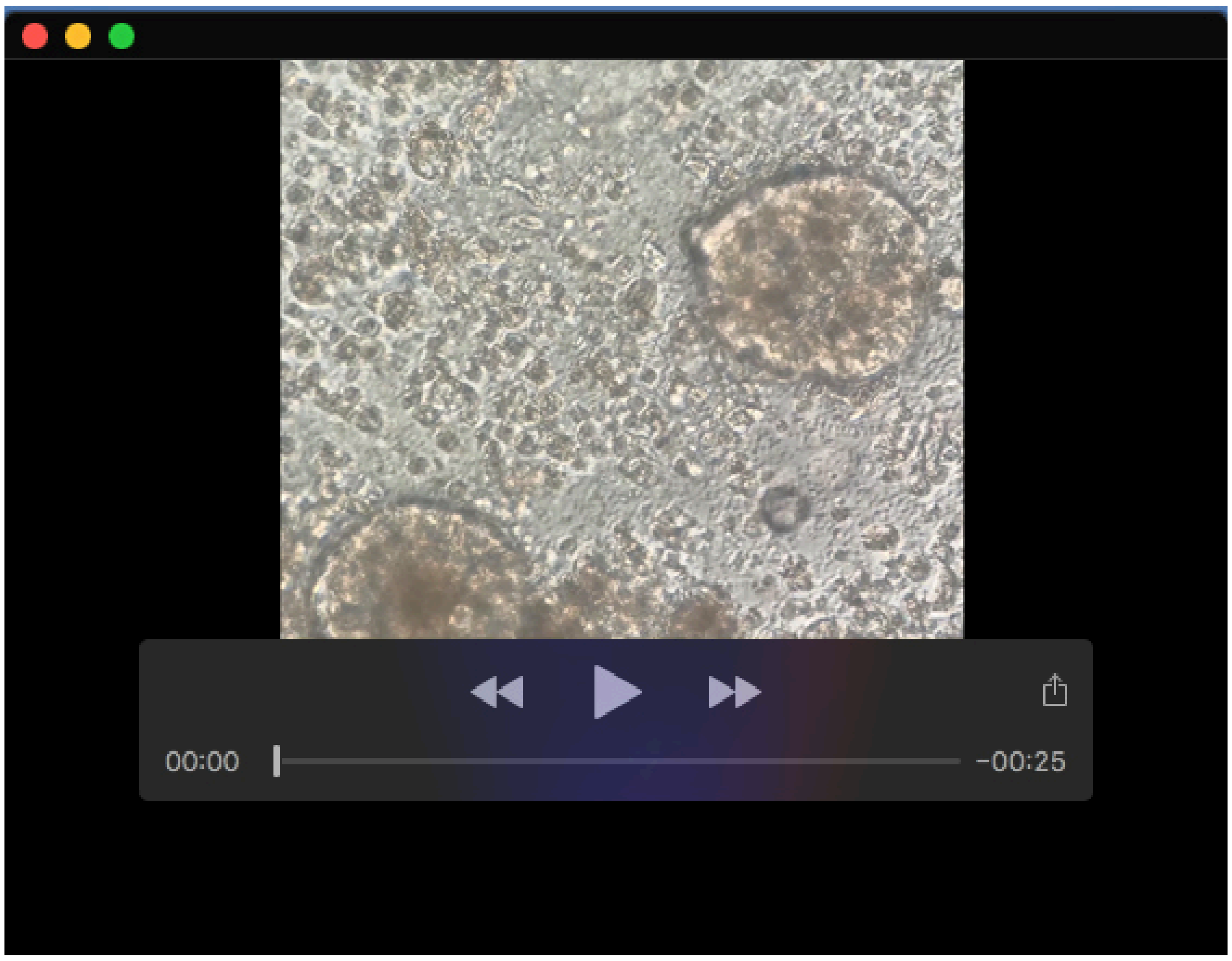

Movie 1. Ciliary beat frequency of organoid at 4 weeks of cultures derived from CRS patients.
